# Supplementary material for: Human influenza A virus H1N1 in marine mammals in California, 2019
Source: PLoS One. 2023 Mar 30;18(3):e0283049. doi: 10.1371/journal.pone.0283049 (PMC10062622; doi:10.1371/journal.pone.0283049)
Supplement: S1 Table — (DOCX) [file pone.0283049.s001.docx]

| **Number** | **GISAID ID** | **Isolate Name** | **Clade/Subclade** | **Collection Date** | **Segment** |
| --- | --- | --- | --- | --- | --- |
| 1 | EPI_ISL_31158 | A/California/07/2009 | clade1 | 2009-04-09 | all 8 segments |
| 2 | EPI_ISL_94764 | A/Mexico/2208/2011 | clade2 | 2011-03-15 | HA |
| 3 | EPI_ISL_99939 | A/Hong_Kong/3960/2011 | clade2 | 2011-04-21 | HA |
| 4 | EPI_ISL_93746 | A/Hong_Kong/3934/2011 | clade3 | 2011-03-29 | HA |
| 5 | EPI_ISL_79722 | A/Christchurch/16/2010 | clade4 | 2010-07-12 | HA |
| 6 | EPI_ISL_79719 | A/Brisbane/10/2010 | clade4 | 2010-04-29 | HA; NA |
| 7 | EPI_ISL_90787 | A/Astrakhan/1/2011 | clade5 | 2011-02-28 | HA; NA |
| 8 | EPI_ISL_90760 | A/St.Petersburg/27/2011 | clade6 | 2011-02-14 | HA; NA |
| 9 | EPI_ISL_89916 | A/St.Petersburg/100/2011 | clade7 | 2011-03-14 | HA; NA |
| 10 | EPI_ISL_127652 | A/Hong_Kong/5659/2012 | 6A | 2012-05-21 | HA; NA |
| 11 | EPI_ISL_145448 | A/South_Africa/3626/2013 | 6B | 2013-06-06 | all 8 segments |
| 12 | EPI_ISL_315701 | A/Dnipro/409/2018 | 6B.1 | 2018-04-03 | HA |
| 13 | EPI_ISL_282309 | A/Victoria/55/2017 | 6B.1 | 2017-07-18 | NA |
| 14 | EPI_ISL_336680 | A/Michigan/45/2015 | 6B.1 | 2015-09-07 | all 8 segments |
| 15 | EPI_ISL_284660 | A/Paris/1447/2017 | 6B.1A | 2017-10-20 | all 8 segments |
| 16 | EPI_ISL_315812 | A/Switzerland/2656/2017 | 6B.1A | 2017-12-21 | all 8 segments |
| 17 | EPI_ISL_290749 | A/Tennessee/61/2017 | 6B.1A | 2017-11-29 | HA |
| 18 | EPI_ISL_344858 | A/Brisbane/02/2018 | 6B.1A.1 | 2018-01-04 | all 8 segments |
| 19 | EPI_ISL_364843 | A/Denmark/2728/2019 | 6B.1A.2 | 2019-03-19 | HA |
| 20 | EPI_ISL_347404 | A/Norway/3737/2018 | 6B.1A.3 | 2018-11-27 | HA |
| 21 | EPI_ISL_347329 | A/Schleswig-Holstein/3/2018 | 6B.1A.4 | 1905-07-10 | HA |
| 22 | EPI_ISL_332840 | A/Norway/3433/2018 | 6B.1A.5a | 2018-10-30 | HA |
| 23 | EPI_ISL_471342 | A/Saint-Petersburg/RII-4304S/2020 | 6B.1A.5a | 2020-03-26 | all 8 segments |
| 24 | EPI_ISL_397020 | A/Hawaii/66/2019 | 6B.1a.5a | 2019-09-19 | HA |
| 25 | EPI_ISL_401203 | A/Maryland/42/2019 | 6B.1A.5b | 2019-11-29 | all 8 segments |
| 26 | EPI_ISL_613417 | A/Iowa/22/2020 | 6B.1A.5b | 2020-09-24 | HA |
| 27 | EPI_ISL_463730 | A/Catalonia/NSVH101183778/2020 | 6B.1A.5b | 2020-02-01 | all 8 segments |
| 28 | EPI_ISL_294121 | A/Switzerland/3330/2017 | 6B.1A.5b | 2017-12-20 | HA |
| 29 | EPI_ISL_4005762 | A/Ghana/64/2021 | 6B.1A.5a.1 | 2021-04-26 | all 8 segments |
| 30 | EPI_ISL_4005757 | A/Ghana/167/2021 | 6B.1A.5a.1 | 2021-07-01 | all 8 segments |
| 31 | EPI_ISL_377080 | A/Guangdong-Maonan/SWL1536/2019 | 6B.1A.5a.1 | 2019-06-17 | all 8 segments |
| 32 | EPI_ISL_397028 | A/Hawaii/70/2019 | 6B.1A.5a.1 | 2019-10-05 | all 8 segments |
| 33 | EPI_ISL_1459510 | A/Iowa/01/2021 | 6B.1A.5a.1 | 2021-02-24 | all 8 segments |
| 34 | EPI_ISL_1296414 | A/Nigeria/5065/2020 | 6B.1A.5a.1 | 2020-12-01 | all 8 segments |
| 35 | EPI_ISL_404460 | A/Wisconsin/588/2019 | 6B.1A.5a.2 | 2019-12-19 | all 8 segments |
| 36 | EPI_ISL_3376993 | A/India/Pun-NIV325329/2021 | 6B.1A.5a.2 | 2021-06-28 | HA |
| 37 | EPI_ISL_338060 | A/Ireland/84630/2018 | 6B.1A.6 | 2018-11-28 | HA |
| 38 | EPI_ISL_410792 | A/Louisiana/01/2020 | 6B.1A.7 | 2020-01-04 | all 8 segments |
| 39 | EPI_ISL_357803 | A/Slovenia/1489/2019 | 6B.1A.7 | 2019-03-11 | HA |
| 40 | EPI_ISL_205291 | A/Iowa/53/2015 | 6B.2 | 2015-11-04 | all 8 segments |
| 41 | EPI_ISL_165501 | A/Dakar/02/2014 | 6C | 2014-01-23 | HA; NA |
