# Supplementary material for: Human influenza A virus H1N1 in marine mammals in California, 2019
Source: PLoS One. 2023 Mar 30;18(3):e0283049. doi: 10.1371/journal.pone.0283049 (PMC10062622; doi:10.1371/journal.pone.0283049)
Supplement: S1 File — The sequence in dark blue is the IAV genome from a Northern elephant seal in this study. Green sequences are IAV from other seals not part of this study. H1N1pdm09 subclade 6B.1A.1 represented by the reference 2019–2020 vaccine virus A/Brisbane/02/2018 is highlighted in yellow; the clade 6B.1A –in blue. Bootstrap supports are indicated next to the nodes, while branch lengths are scaled according to the number of nucleotide substitutions per site. (PDF) [file pone.0283049.s002.pdf]

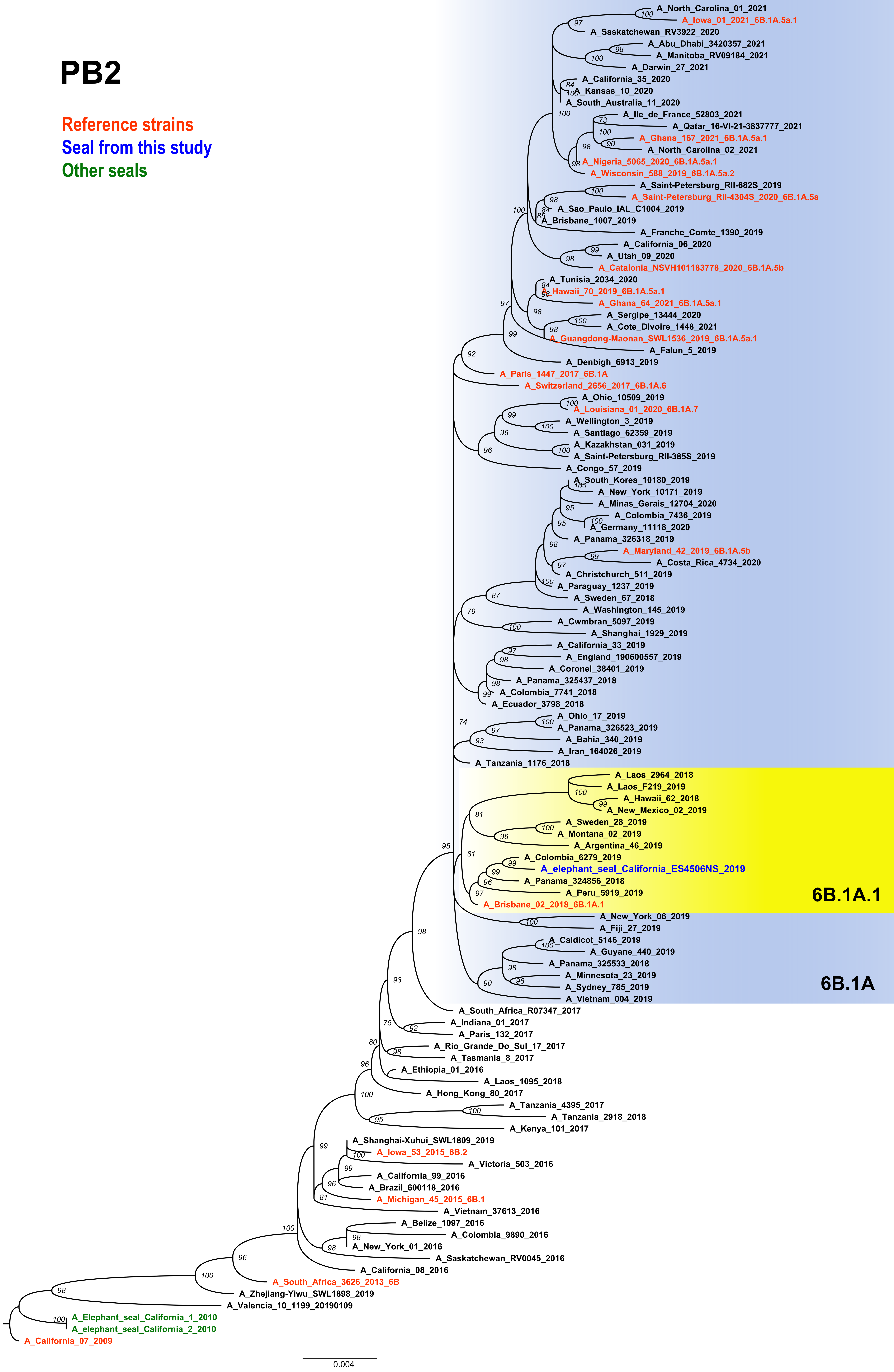

PB2
Reference strains
Seal from this study
Other seals
A_North_Carolina_01_2021
A_Iowa_01_2021_6B.1A.5a.1
A_Saskatchewan_RV3922_2020
A_Abu_Dhabi_3420357_2021
A_Manitoba_RV09184_2021
A_Darwin_27_2021
A_California_35_2020
A_Kansas_10_2020
A_South_Australia_11_2020
A_Ile_de_France_52803_2021
A_Qatar_16-VI-21-3837777_2021
A_Ghana_167_2021_6B.1A.5a.1
A_North_Carolina_02_2021
A_Nigeria_5065_2020_6B.1A.5a.1
A_Wisconsin_588_2019_6B.1A.5a.2
A_Saint-Petersburg_RII-682S_2019
A_Saint-Petersburg_RII-4304S_2020_6B.1A.5a
A_Sao_Paulo_IAL_C1004_2019
A_Brisbane_1007_2019
A_Franche_Comte_1390_2019
A_California_06_2020
A_Utah_09_2020
A_Catalonia_NSVH101183778_2020_6B.1A.5b
A_Tunisia_2034_2020
A_Hawaii_70_2019_6B.1A.5a.1
A_Ghana_64_2021_6B.1A.5a.1
A_Sergipe_13444_2020
A_Cote_DIvoire_1448_2021
A_Guangdong-Maonan_SWL1536_2019_6B.1A.5a.1
A_Falun_5_2019
A_Denbigh_6913_2019
A_Paris_1447_2017_6B.1A
A_Switzerland_2656_2017_6B.1A.6
A_Ohio_10509_2019
A_Louisiana_01_2020_6B.1A.7
A_Wellington_3_2019
A_Santiago_62359_2019
A_Kazakhstan_031_2019
A_Saint-Petersburg_RII-385S_2019
A_Congo_57_2019
A_South_Korea_10180_2019
A_New_York_10171_2019
A_Minas_Gerais_12704_2020
A_Colombia_7436_2019
A_Germany_11118_2020
A_Panama_326318_2019
A_Maryland_42_2019_6B.1A.5b
A_Costa_Rica_4734_2020
A_Christchurch_511_2019
A_Paraguay_1237_2019
A_Sweden_67_2018
A_Washington_145_2019
A_Cwmbran_5097_2019
A_Shanghai_1929_2019
A_California_33_2019
A_England_190600557_2019
A_Coronel_38401_2019
A_Panama_325437_2018
A_Colombia_7741_2018
A_Ecuador_3798_2018
A_Ohio_17_2019
A_Panama_326523_2019
A_Bahia_340_2019
A_Iran_164026_2019
A_Tanzania_1176_2018
A_Laos_2964_2018
A_Laos_F219_2019
A_Hawaii_62_2018
A_New_Mexico_02_2019
A_Sweden_28_2019
A_Montana_02_2019
A_Argentina_46_2019
A_Colombia_6279_2019
A_elephant_seal_California_ES4506NS_2019
A_Panama_324856_2018
A_Peru_5919_2019
A_Brisbane_02_2018_6B.1A.1
6B.1A.1
A_New_York_06_2019
A_Fiji_27_2019
A_Caldicot_5146_2019
A_Guyane_440_2019
A_Panama_325533_2018
A_Minnesota_23_2019
A_Sydney_785_2019
A_Vietnam_004_2019
6B.1A
A_South_Africa_R07347_2017
A_Indiana_01_2017
A_Paris_132_2017
A_Rio_Grande_Do_Sul_17_2017
A_Tasmania_8_2017
A_Ethiopia_01_2016
A_Laos_1095_2018
A_Hong_Kong_80_2017
A_Tanzania_4395_2017
A_Tanzania_2918_2018
A_Kenya_101_2017
A_Shanghai-Xuhui_SWL1809_2019
A_Iowa_53_2015_6B.2
A_Victoria_503_2016
A_California_99_2016
A_Brazil_600118_2016
A_Michigan_45_2015_6B.1
A_Vietnam_37613_2016
A_Belize_1097_2016
A_Colombia_9890_2016
A_New_York_01_2016
A_Saskatchewan_RV0045_2016
A_California_08_2016
A_South_Africa_3626_2013_6B
A_Zhejiang-Yiwu_SWL1898_2019
A_Valencia_10_1199_20190109
A_Elephant_seal_California_1_2010
A_elephant_seal_California_2_2010
A_California_07_2009
0.004

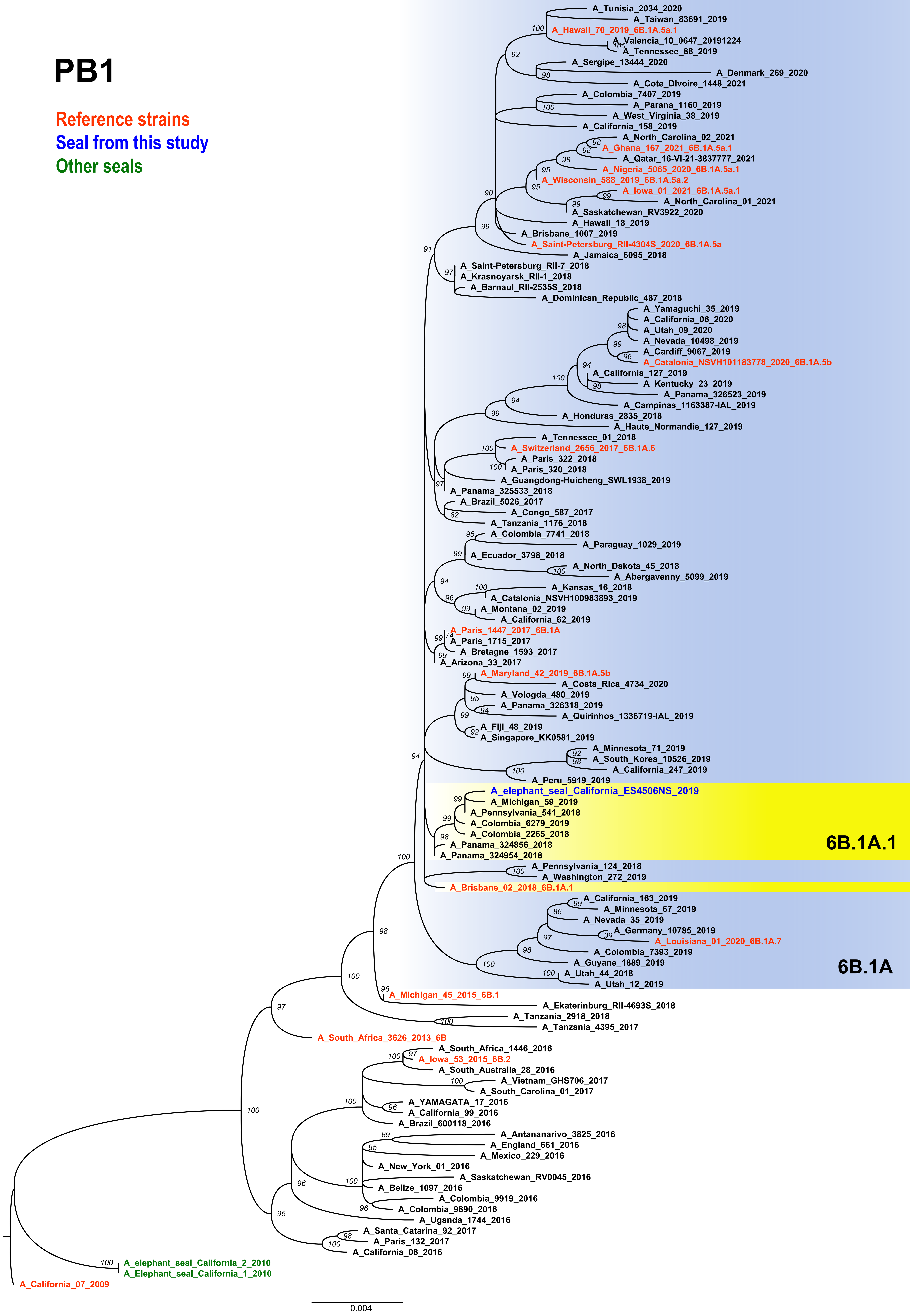
PB1
Reference strains
Seal from this study
Other seals
A_Tunisia_2034_2020
A_Taiwan_83691_2019
A_Hawaii_70_2019_6B.1A.5a.1
A_Valencia_10_0647_20191224
A_Tennessee_88_2019
A_Sergipe_13444_2020
A_Denmark_269_2020
A_Cote_DIvoire_1448_2021
A_Colombia_7407_2019
A_Parana_1160_2019
A_West_Virginia_38_2019
A_California_158_2019
A_North_Carolina_02_2021
A_Ghana_167_2021_6B.1A.5a.1
A_Qatar_16-VI-21-3837777_2021
A_Nigeria_5065_2020_6B.1A.5a.1
A_Wisconsin_588_2019_6B.1A.5a.2
A_Iowa_01_2021_6B.1A.5a.1
A_North_Carolina_01_2021
A_Saskatchewan_RV3922_2020
A_Hawaii_18_2019
A_Brisbane_1007_2019
A_Saint-Petersburg_RII-4304S_2020_6B.1A.5a
A_Jamaica_6095_2018
A_Saint-Petersburg_RII-7_2018
A_Krasnoyarsk_RII-1_2018
A_Barnaul_RII-2535S_2018
A_Dominican_Republic_487_2018
A_Yamaguchi_35_2019
A_California_06_2020
A_Utah_09_2020
A_Nevada_10498_2019
A_Cardiff_9067_2019
A_Catalonia_NSVH101183778_2020_6B.1A.5b
A_California_127_2019
A_Kentucky_23_2019
A_Panama_326523_2019
A_Campinas_1163387-IAL_2019
A_Honduras_2835_2018
A_Haute_Normandie_127_2019
A_Tennessee_01_2018
A_Switzerland_2656_2017_6B.1A.6
A_Paris_322_2018
A_Paris_320_2018
A_Guangdong-Huicheng_SWL1938_2019
A_Panama_325533_2018
A_Brazil_5026_2017
A_Congo_587_2017
A_Tanzania_1176_2018
A_Colombia_7741_2018
A_Paraguay_1029_2019
A_Ecuador_3798_2018
A_North_Dakota_45_2018
A_Abergavenny_5099_2019
A_Kansas_16_2018
A_Catalonia_NSVH100983893_2019
A_Montana_02_2019
A_California_62_2019
A_Paris_1447_2017_6B.1A
A_Paris_1715_2017
A_Bretagne_1593_2017
A_Arizona_33_2017
A_Maryland_42_2019_6B.1A.5b
A_Costa_Rica_4734_2020
A_Vologda_480_2019
A_Panama_326318_2019
A_Quirinhos_1336719-IAL_2019
A_Fiji_48_2019
A_Singapore_KK0581_2019
A_Minnesota_71_2019
A_South_Korea_10526_2019
A_California_247_2019
A_Peru_5919_2019
A_elephant_seal_California_ES4506NS_2019
A_Michigan_59_2019
A_Pennsylvania_541_2018
A_Colombia_6279_2019
A_Colombia_2265_2018
A_Panama_324856_2018
A_Panama_324954_2018
6B.1A.1
A_Pennsylvania_124_2018
A_Washington_272_2019
A_Brisbane_02_2018_6B.1A.1
A_California_163_2019
A_Minnesota_67_2019
A_Nevada_35_2019
A_Germany_10785_2019
A_Louisiana_01_2020_6B.1A.7
A_Colombia_7393_2019
A_Guyane_1889_2019
A_Utah_44_2018
A_Utah_12_2019
6B.1A
A_Michigan_45_2015_6B.1
A_Ekaterinburg_RII-4693S_2018
A_Tanzania_2918_2018
A_Tanzania_4395_2017
A_South_Africa_3626_2013_6B
A_South_Africa_1446_2016
A_Iowa_53_2015_6B.2
A_South_Australia_28_2016
A_Vietnam_GHS706_2017
A_South_Carolina_01_2017
A_YAMAGATA_17_2016
A_California_99_2016
A_Brazil_600118_2016
A_Antananarivo_3825_2016
A_England_661_2016
A_Mexico_229_2016
A_New_York_01_2016
A_Saskatchewan_RV0045_2016
A_Belize_1097_2016
A_Colombia_9919_2016
A_Colombia_9890_2016
A_Uganda_1744_2016
A_Santa_Catarina_92_2017
A_Paris_132_2017
A_California_08_2016
A_elephant_seal_California_2_2010
A_Elephant_seal_California_1_2010
A_California_07_2009
0.004

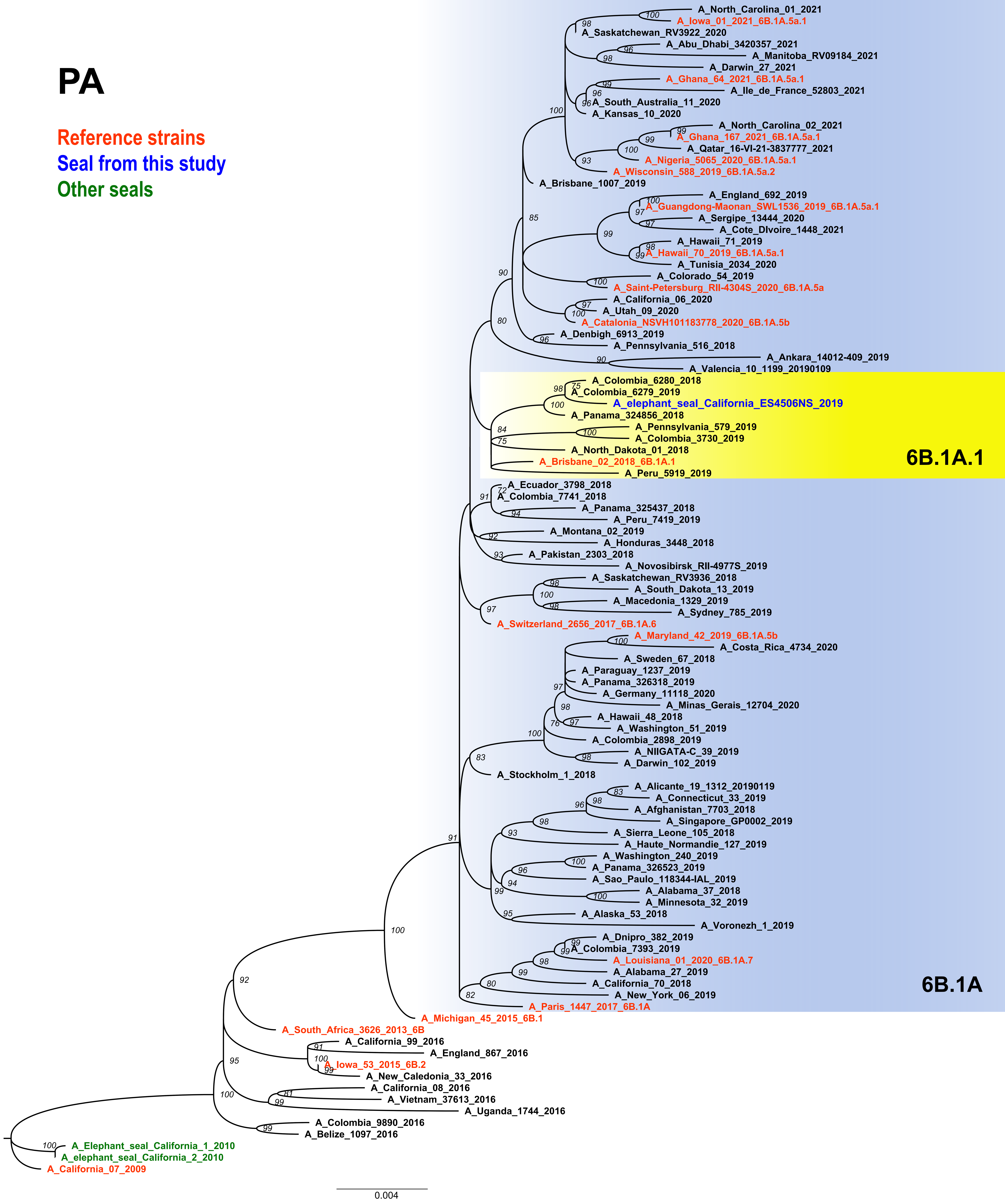

PA
Reference strains
Seal from this study
Other seals
A_North_Carolina_01_2021
A_Iowa_01_2021_6B.1A.5a.1
A_Saskatchewan_RV3922_2020
A_Abu_Dhabi_3420357_2021
A_Manitoba_RV09184_2021
A_Darwin_27_2021
A_Ghana_64_2021_6B.1A.5a.1
A_Ile_de_France_52803_2021
A_South_Australia_11_2020
A_Kansas_10_2020
A_North_Carolina_02_2021
A_Ghana_167_2021_6B.1A.5a.1
A_Qatar_16-VI-21-3837777_2021
A_Nigeria_5065_2020_6B.1A.5a.1
A_Wisconsin_588_2019_6B.1A.5a.2
A_Brisbane_1007_2019
A_England_692_2019
A_Guangdong-Maonan_SWL1536_2019_6B.1A.5a.1
A_Sergipe_13444_2020
A_Cote_DIvoire_1448_2021
A_Hawaii_71_2019
A_Hawaii_70_2019_6B.1A.5a.1
A_Tunisia_2034_2020
A_Colorado_54_2019
A_Saint-Petersburg_RII-4304S_2020_6B.1A.5a
A_California_06_2020
A_Utah_09_2020
A_Catalonia_NSVH101183778_2020_6B.1A.5b
A_Denbigh_6913_2019
A_Pennsylvania_516_2018
A_Ankara_14012-409_2019
A_Valencia_10_1199_20190109
A_Colombia_6280_2018
A_Colombia_6279_2019
A_elephant_seal_California_ES4506NS_2019
A_Panama_324856_2018
A_Pennsylvania_579_2019
A_Colombia_3730_2019
A_North_Dakota_01_2018
A_Brisbane_02_2018_6B.1A.1
A_Peru_5919_2019
6B.1A.1
A_Ecuador_3798_2018
A_Colombia_7741_2018
A_Panama_325437_2018
A_Peru_7419_2019
A_Montana_02_2019
A_Honduras_3448_2018
A_Pakistan_2303_2018
A_Novosibirsk_RII-4977S_2019
A_Saskatchewan_RV3936_2018
A_South_Dakota_13_2019
A_Macedonia_1329_2019
A_Sydney_785_2019
A_Switzerland_2656_2017_6B.1A.6
A_Maryland_42_2019_6B.1A.5b
A_Costa_Rica_4734_2020
A_Sweden_67_2018
A_Paraguay_1237_2019
A_Panama_326318_2019
A_Germany_11118_2020
A_Minas_Gerais_12704_2020
A_Hawaii_48_2018
A_Washington_51_2019
A_Colombia_2898_2019
A_NIIGATA-C_39_2019
A_Darwin_102_2019
A_Stockholm_1_2018
A_Alicante_19_1312_20190119
A_Connecticut_33_2019
A_Afghanistan_7703_2018
A_Singapore_GP0002_2019
A_Sierra_Leone_105_2018
A_Haute_Normandie_127_2019
A_Washington_240_2019
A_Panama_326523_2019
A_Sao_Paulo_118344-IAL_2019
A_Alabama_37_2018
A_Minnesota_32_2019
A_Alaska_53_2018
A_Voronezh_1_2019
A_Dnipro_382_2019
A_Colombia_7393_2019
A_Louisiana_01_2020_6B.1A.7
A_Alabama_27_2019
A_California_70_2018
A_New_York_06_2019
A_Paris_1447_2017_6B.1A
6B.1A
A_Michigan_45_2015_6B.1
A_South_Africa_3626_2013_6B
A_California_99_2016
A_England_867_2016
A_Iowa_53_2015_6B.2
A_New_Caledonia_33_2016
A_California_08_2016
A_Vietnam_37613_2016
A_Uganda_1744_2016
A_Colombia_9890_2016
A_Belize_1097_2016
A_Elephant_seal_California_1_2010
A_elephant_seal_California_2_2010
A_California_07_2009
0.004

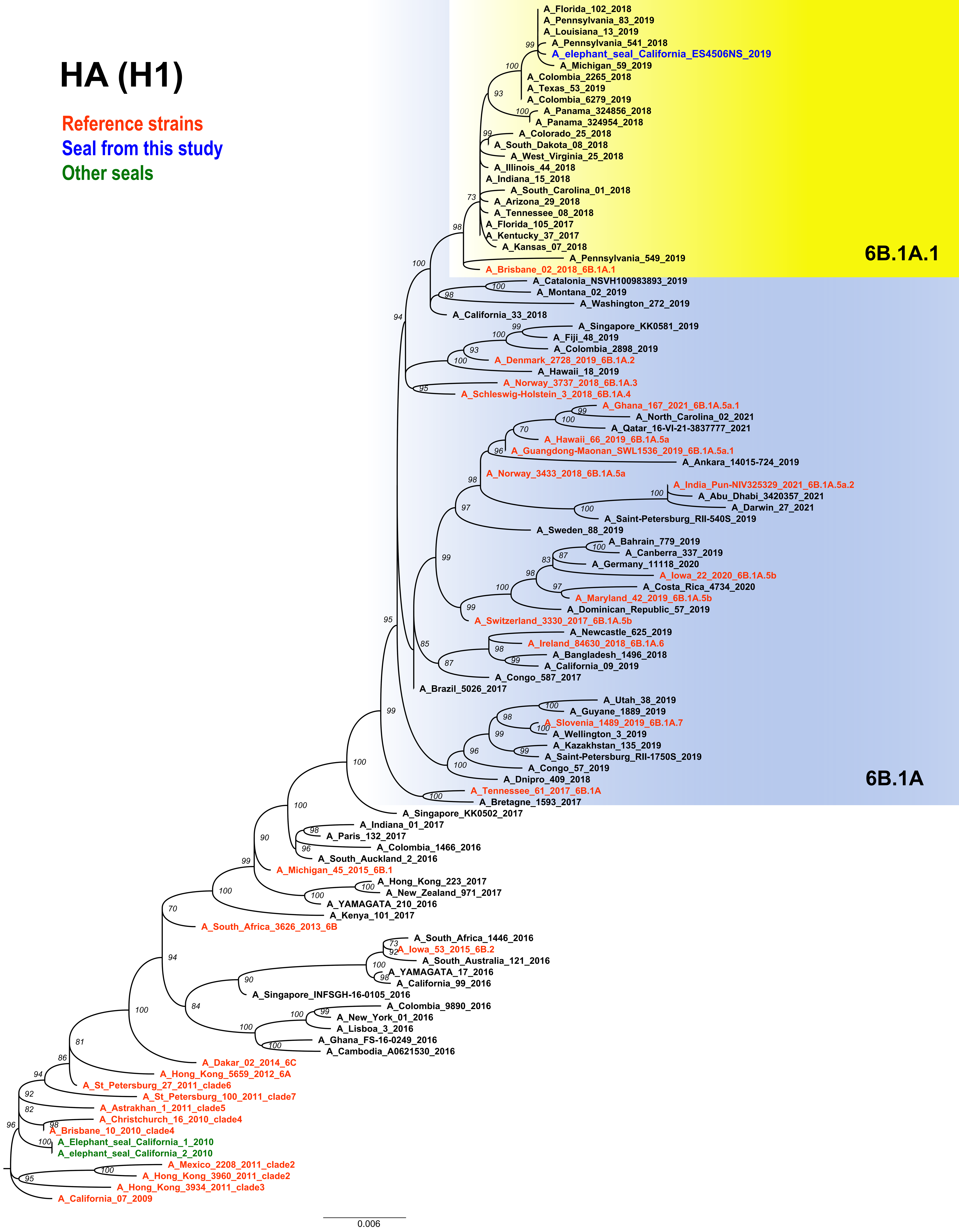

HA (H1)
Reference strains
Seal from this study
Other seals
A_Florida_102_2018
A_Pennsylvania_83_2019
A_Louisiana_13_2019
A_Pennsylvania_541_2018
A_elephant_seal_California_ES4506NS_2019
A_Michigan_59_2019
A_Colombia_2265_2018
A_Texas_53_2019
A_Colombia_6279_2019
A_Panama_324856_2018
A_Panama_324954_2018
A_Colorado_25_2018
A_South_Dakota_08_2018
A_West_Virginia_25_2018
A_Illinois_44_2018
A_Indiana_15_2018
A_South_Carolina_01_2018
A_Arizona_29_2018
A_Tennessee_08_2018
A_Florida_105_2017
A_Kentucky_37_2017
A_Kansas_07_2018
A_Pennsylvania_549_2019
A_Brisbane_02_2018_6B.1A.1
6B.1A.1
A_Catalonia_NSVH100983893_2019
A_Montana_02_2019
A_Washington_272_2019
A_California_33_2018
A_Singapore_KK0581_2019
A_Fiji_48_2019
A_Colombia_2898_2019
A_Denmark_2728_2019_6B.1A.2
A_Hawaii_18_2019
A_Norway_3737_2018_6B.1A.3
A_Schleswig-Holstein_3_2018_6B.1A.4
A_Ghana_167_2021_6B.1A.5a.1
A_North_Carolina_02_2021
A_Qatar_16-VI-21-3837777_2021
A_Hawaii_66_2019_6B.1A.5a
A_Guangdong-Maonan_SWL1536_2019_6B.1A.5a.1
A_Ankara_14015-724_2019
A_Norway_3433_2018_6B.1A.5a
A_India_Pun-NIV325329_2021_6B.1A.5a.2
A_Abu_Dhabi_3420357_2021
A_Darwin_27_2021
A_Saint-Petersburg_RII-540S_2019
A_Sweden_88_2019
A_Bahrain_779_2019
A_Canberra_337_2019
A_Germany_11118_2020
A_Iowa_22_2020_6B.1A.5b
A_Costa_Rica_4734_2020
A_Maryland_42_2019_6B.1A.5b
A_Dominican_Republic_57_2019
A_Switzerland_3330_2017_6B.1A.5b
A_Newcastle_625_2019
A_Ireland_84630_2018_6B.1A.6
A_Bangladesh_1496_2018
A_California_09_2019
A_Congo_587_2017
A_Brazil_5026_2017
A_Utah_38_2019
A_Guyane_1889_2019
A_Slovenia_1489_2019_6B.1A.7
A_Wellington_3_2019
A_Kazakhstan_135_2019
A_Saint-Petersburg_RII-1750S_2019
A_Congo_57_2019
A_Dnipro_409_2018
6B.1A
A_Tennessee_61_2017_6B.1A
A_Bretagne_1593_2017
A_Singapore_KK0502_2017
A_Indiana_01_2017
A_Paris_132_2017
A_Colombia_1466_2016
A_South_Auckland_2_2016
A_Michigan_45_2015_6B.1
A_Hong_Kong_223_2017
A_New_Zealand_971_2017
A_YAMAGATA_210_2016
A_Kenya_101_2017
A_South_Africa_3626_2013_6B
A_South_Africa_1446_2016
A_Iowa_53_2015_6B.2
A_South_Australia_121_2016
A_YAMAGATA_17_2016
A_California_99_2016
A_Singapore_INFSGH-16-0105_2016
A_Colombia_9890_2016
A_New_York_01_2016
A_Lisboa_3_2016
A_Ghana_FS-16-0249_2016
A_Cambodia_A0621530_2016
A_Dakar_02_2014_6C
A_Hong_Kong_5659_2012_6A
A_St_Petersburg_27_2011_clade6
A_St_Petersburg_100_2011_clade7
A_Astrakhan_1_2011_clade5
A_Christchurch_16_2010_clade4
A_Brisbane_10_2010_clade4
A_Elephant_seal_California_1_2010
A_elephant_seal_California_2_2010
A_Mexico_2208_2011_clade2
A_Hong_Kong_3960_2011_clade2
A_Hong_Kong_3934_2011_clade3
A_California_07_2009
0.006

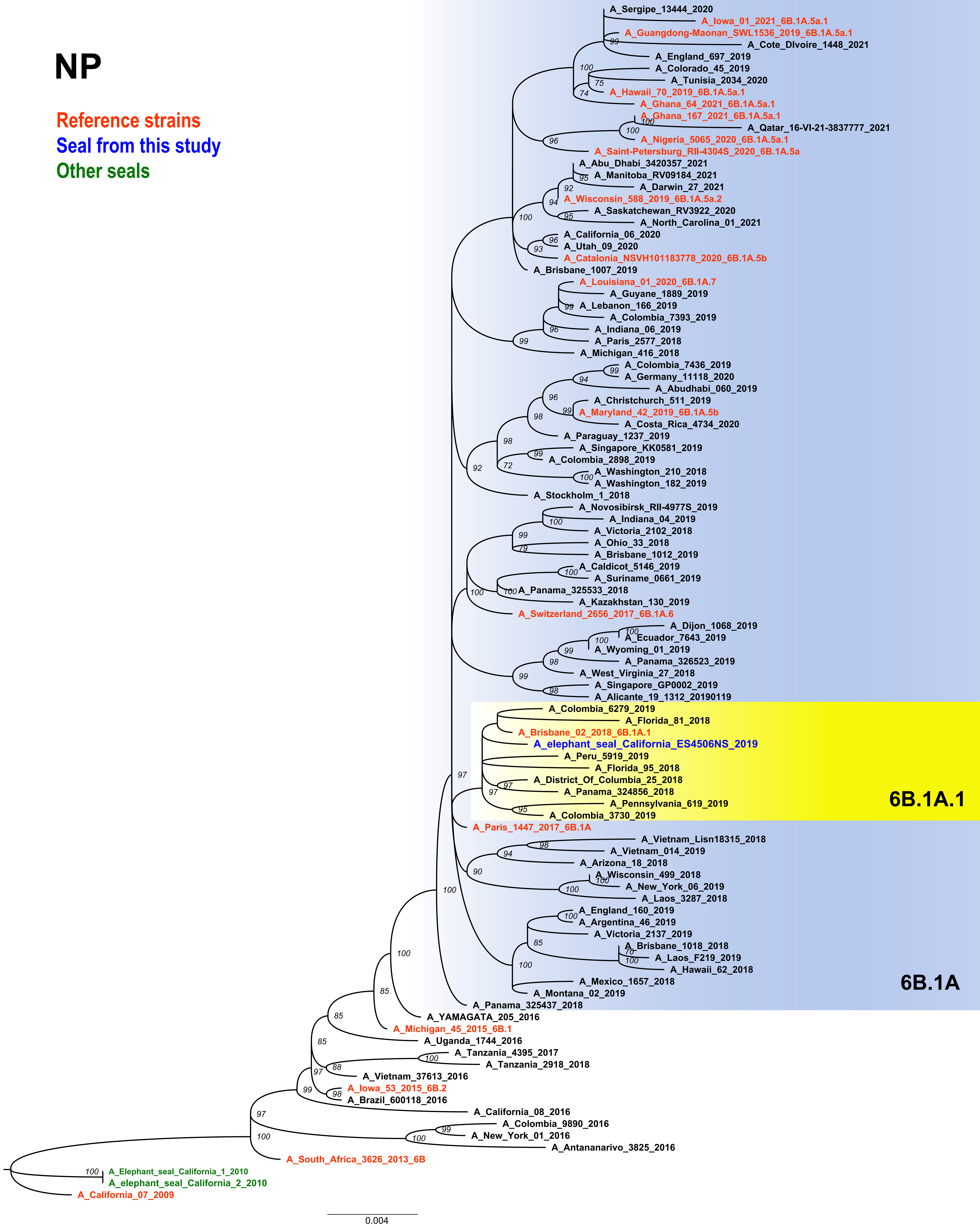

NP
Reference strains
Seal from this study
Other seals
A_Sergipe_13444_2020
A_Iowa_01_2021_6B.1A.5a.1
A_Guangdong-Maonan_SWL1536_2019_6B.1A.5a.1
A_Cote_DIvoire_1448_2021
A_England_697_2019
A_Colorado_45_2019
A_Tunisia_2034_2020
A_Hawaii_70_2019_6B.1A.5a.1
A_Ghana_64_2021_6B.1A.5a.1
A_Ghana_167_2021_6B.1A.5a.1
A_Qatar_16-VI-21-3837777_2021
A_Nigeria_5065_2020_6B.1A.5a.1
A_Saint-Petersburg_RII-4304S_2020_6B.1A.5a
A_Abu_Dhabi_3420357_2021
A_Manitoba_RV09184_2021
A_Darwin_27_2021
A_Wisconsin_588_2019_6B.1A.5a.2
A_Saskatchewan_RV3922_2020
A_North_Carolina_01_2021
A_California_06_2020
A_Utah_09_2020
A_Catalonia_NSVH101183778_2020_6B.1A.5b
A_Brisbane_1007_2019
A_Louisiana_01_2020_6B.1A.7
A_Guyane_1889_2019
A_Lebanon_166_2019
A_Colombia_7393_2019
A_Indiana_06_2019
A_Paris_2577_2018
A_Michigan_416_2018
A_Colombia_7436_2019
A_Germany_11118_2020
A_Abudhabi_060_2019
A_Christchurch_511_2019
A_Maryland_42_2019_6B.1A.5b
A_Costa_Rica_4734_2020
A_Paraguay_1237_2019
A_Singapore_KK0581_2019
A_Colombia_2898_2019
A_Washington_210_2018
A_Washington_182_2019
A_Stockholm_1_2018
A_Novosibirsk_RII-4977S_2019
A_Indiana_04_2019
A_Victoria_2102_2018
A_Ohio_33_2018
A_Brisbane_1012_2019
A_Caldicot_5146_2019
A_Suriname_0661_2019
A_Panama_325533_2018
A_Kazakhstan_130_2019
A_Switzerland_2656_2017_6B.1A.6
A_Dijon_1068_2019
A_Ecuador_7643_2019
A_Wyoming_01_2019
A_Panama_326523_2019
A_West_Virginia_27_2018
A_Singapore_GP0002_2019
A_Alicante_19_1312_20190119
A_Colombia_6279_2019
A_Florida_81_2018
A_Brisbane_02_2018_6B.1A.1
A_elephant_seal_California_ES4506NS_2019
A_Peru_5919_2019
A_Florida_95_2018
A_District_Of_Columbia_25_2018
A_Panama_324856_2018
A_Pennsylvania_619_2019
A_Colombia_3730_2019
6B.1A.1
A_Paris_1447_2017_6B.1A
A_Vietnam_Lisn18315_2018
A_Vietnam_014_2019
A_Arizona_18_2018
A_Wisconsin_499_2018
A_New_York_06_2019
A_Laos_3287_2018
A_England_160_2019
A_Argentina_46_2019
A_Victoria_2137_2019
A_Brisbane_1018_2018
A_Laos_F219_2019
A_Hawaii_62_2018
A_Mexico_1657_2018
A_Montana_02_2019
6B.1A
A_Panama_325437_2018
A_YAMAGATA_205_2016
A_Michigan_45_2015_6B.1
A_Uganda_1744_2016
A_Tanzania_4395_2017
A_Tanzania_2918_2018
A_Vietnam_37613_2016
A_Iowa_53_2015_6B.2
A_Brazil_600118_2016
A_California_08_2016
A_Colombia_9890_2016
A_New_York_01_2016
A_Antananarivo_3825_2016
A_South_Africa_3626_2013_6B
A_Elephant_seal_California_1_2010
A_elephant_seal_California_2_2010
A_California_07_2009
0.004

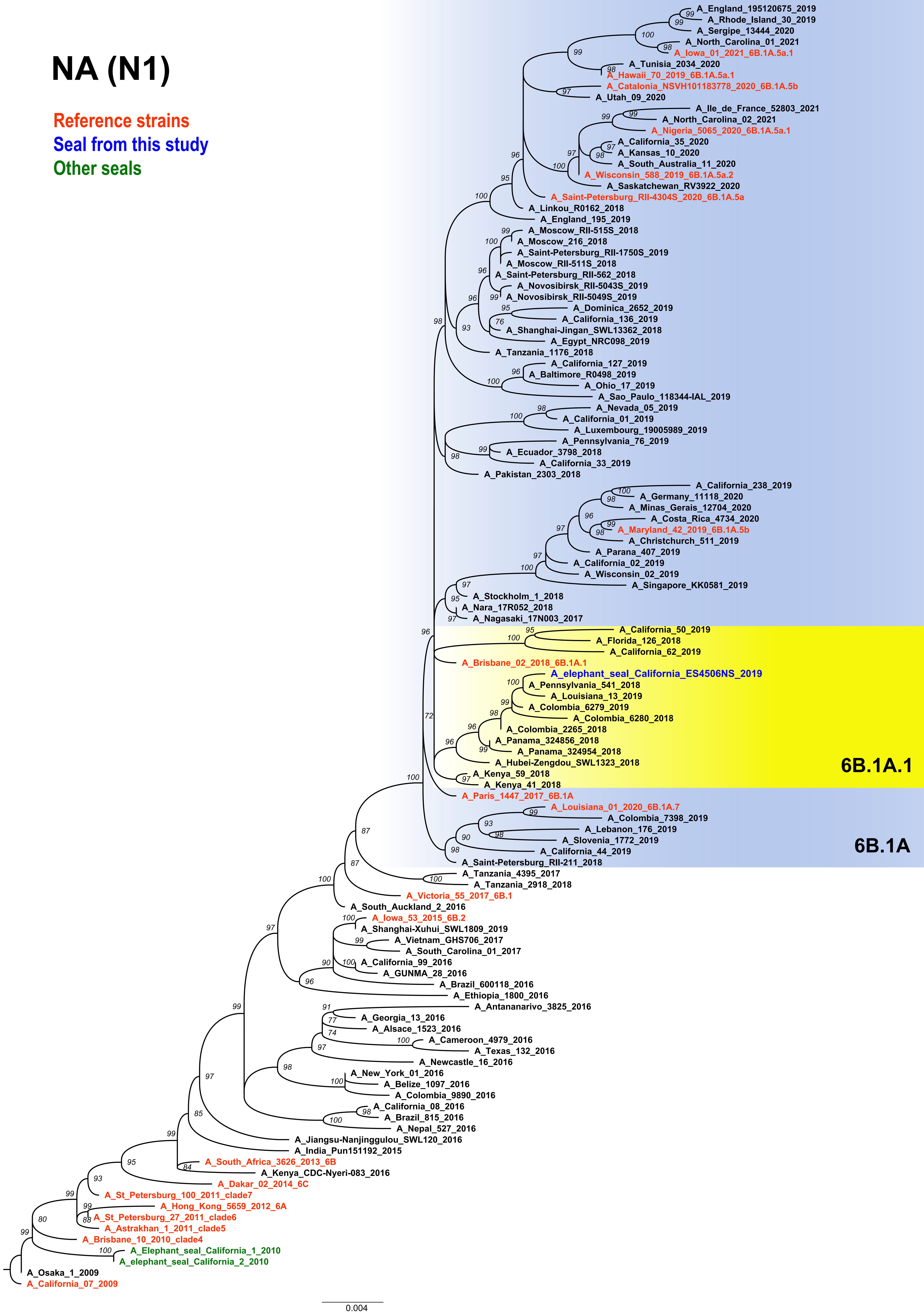
NA (N1)
Reference strains
Seal from this study
Other seals
A_England_195120675_2019
A_Rhode_Island_30_2019
A_Sergipe_13444_2020
A_North_Carolina_01_2021
A_Iowa_01_2021_6B.1A.5a.1
A_Tunisia_2034_2020
A_Hawaii_70_2019_6B.1A.5a.1
A_Catalonia_NSVH101183778_2020_6B.1A.5b
A_Utah_09_2020
A_Ile_de_France_52803_2021
A_North_Carolina_02_2021
A_Nigeria_5065_2020_6B.1A.5a.1
A_California_35_2020
A_Kansas_10_2020
A_South_Australia_11_2020
A_Wisconsin_588_2019_6B.1A.5a.2
A_Saskatchewan_RV3922_2020
A_Saint-Petersburg_RII-4304S_2020_6B.1A.5a
A_Linkou_R0162_2018
A_England_195_2019
A_Moscow_RII-515S_2018
A_Moscow_216_2018
A_Saint-Petersburg_RII-1750S_2019
A_Moscow_RII-511S_2018
A_Saint-Petersburg_RII-562_2018
A_Novosibirsk_RII-5043S_2019
A_Novosibirsk_RII-5049S_2019
A_Dominica_2652_2019
A_California_136_2019
A_Shanghai-Jingan_SWL13362_2018
A_Egypt_NRC098_2019
A_Tanzania_1176_2018
A_California_127_2019
A_Baltimore_R0498_2019
A_Ohio_17_2019
A_Sao_Paulo_118344-IAL_2019
A_Nevada_05_2019
A_California_01_2019
A_Luxembourg_19005989_2019
A_Pennsylvania_76_2019
A_Ecuador_3798_2018
A_California_33_2019
A_Pakistan_2303_2018
A_California_238_2019
A_Germany_11118_2020
A_Minas_Gerais_12704_2020
A_Costa_Rica_4734_2020
A_Maryland_42_2019_6B.1A.5b
A_Christchurch_511_2019
A_Parana_407_2019
A_California_02_2019
A_Wisconsin_02_2019
A_Singapore_KK0581_2019
A_Stockholm_1_2018
A_Nara_17R052_2018
A_Nagasaki_17N003_2017
A_California_50_2019
A_Florida_126_2018
A_California_62_2019
A_Brisbane_02_2018_6B.1A.1
A_elephant_seal_California_ES4506NS_2019
A_Pennsylvania_541_2018
A_Louisiana_13_2019
A_Colombia_6279_2019
A_Colombia_6280_2018
A_Colombia_2265_2018
A_Panama_324856_2018
A_Panama_324954_2018
A_Hubei-Zengdou_SWL1323_2018
A_Kenya_59_2018
A_Kenya_41_2018
6B.1A.1
A_Paris_1447_2017_6B.1A
A_Louisiana_01_2020_6B.1A.7
A_Colombia_7398_2019
A_Lebanon_176_2019
A_Slovenia_1772_2019
A_California_44_2019
A_Saint-Petersburg_RII-211_2018
6B.1A
A_Tanzania_4395_2017
A_Tanzania_2918_2018
A_Victoria_55_2017_6B.1
A_South_Auckland_2_2016
A_Iowa_53_2015_6B.2
A_Shanghai-Xuhui_SWL1809_2019
A_Vietnam_GHS706_2017
A_South_Carolina_01_2017
A_California_99_2016
A_GUNMA_28_2016
A_Brazil_600118_2016
A_Ethiopia_1800_2016
A_Antananarivo_3825_2016
A_Georgia_13_2016
A_Alsace_1523_2016
A_Cameroon_4979_2016
A_Texas_132_2016
A_Newcastle_16_2016
A_New_York_01_2016
A_Belize_1097_2016
A_Colombia_9890_2016
A_California_08_2016
A_Brazil_815_2016
A_Nepal_527_2016
A_Jiangsu-Nanjinggulou_SWL120_2016
A_India_Pun151192_2015
A_South_Africa_3626_2013_6B
A_Kenya_CDC-Nyeri-083_2016
A_Dakar_02_2014_6C
A_St_Petersburg_100_2011_clade7
A_Hong_Kong_5659_2012_6A
A_St_Petersburg_27_2011_clade6
A_Astrakhan_1_2011_clade5
A_Brisbane_10_2010_clade4
A_Elephant_seal_California_1_2010
A_elephant_seal_California_2_2010
A_Osaka_1_2009
A_California_07_2009
0.004

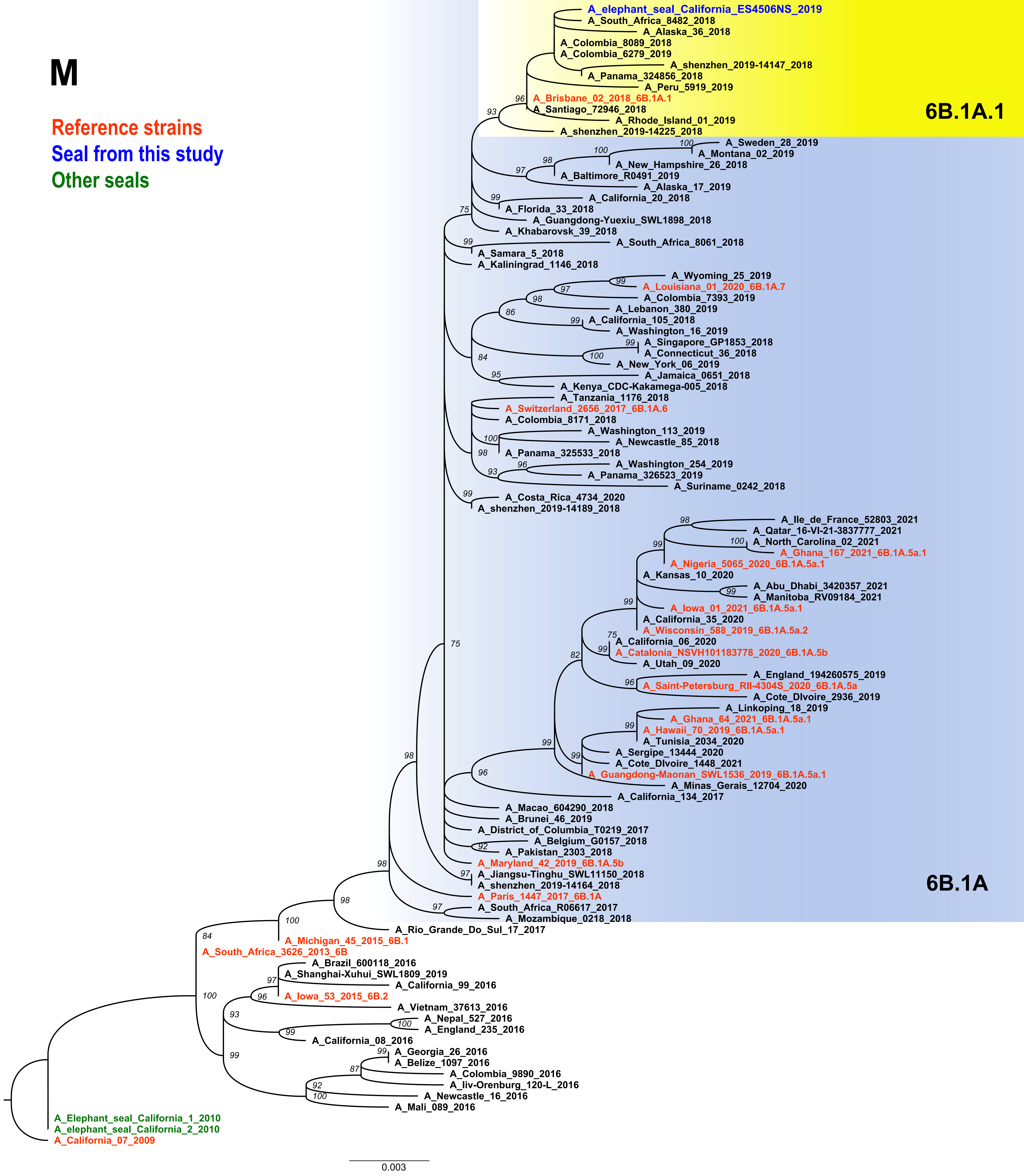
M
Reference strains
Seal from this study
Other seals
6B.1A.1
6B.1A
A_elephant_seal_California_ES4506NS_2019
A_South_Africa_8482_2018
A_Alaska_36_2018
A_Colombia_8089_2018
A_Colombia_6279_2019
A_shenzhen_2019-14147_2018
A_Panama_324856_2018
A_Peru_5919_2019
A_Brisbane_02_2018_6B.1A.1
A_Santiago_72946_2018
A_Rhode_Island_01_2019
A_shenzhen_2019-14225_2018
A_Sweden_28_2019
A_Montana_02_2019
A_New_Hampshire_26_2018
A_Baltimore_R0491_2019
A_Alaska_17_2019
A_California_20_2018
A_Florida_33_2018
A_Guangdong-Yuexiu_SWL1898_2018
A_Khabarovsk_39_2018
A_South_Africa_8061_2018
A_Samara_5_2018
A_Kaliningrad_1146_2018
A_Wyoming_25_2019
A_Louisiana_01_2020_6B.1A.7
A_Colombia_7393_2019
A_Lebanon_380_2019
A_California_105_2018
A_Washington_16_2019
A_Singapore_GP1853_2018
A_Connecticut_36_2018
A_New_York_06_2019
A_Jamaica_0651_2018
A_Kenya_CDC-Kakamega-005_2018
A_Tanzania_1176_2018
A_Switzerland_2656_2017_6B.1A.6
A_Colombia_8171_2018
A_Washington_113_2019
A_Newcastle_85_2018
A_Panama_325533_2018
A_Washington_254_2019
A_Panama_326523_2019
A_Suriname_0242_2018
A_Costa_Rica_4734_2020
A_shenzhen_2019-14189_2018
A_Ile_de_France_52803_2021
A_Qatar_16-VI-21-3837777_2021
A_North_Carolina_02_2021
A_Ghana_167_2021_6B.1A.5a.1
A_Nigeria_5065_2020_6B.1A.5a.1
A_Kansas_10_2020
A_Abu_Dhabi_3420357_2021
A_Manitoba_RV09184_2021
A_Iowa_01_2021_6B.1A.5a.1
A_California_35_2020
A_Wisconsin_588_2019_6B.1A.5a.2
A_California_06_2020
A_Catalonia_NSVH101183778_2020_6B.1A.5b
A_Utah_09_2020
A_England_194260575_2019
A_Saint-Petersburg_RII-4304S_2020_6B.1A.5a
A_Cote_DIvoire_2936_2019
A_Linkoping_18_2019
A_Ghana_64_2021_6B.1A.5a.1
A_Hawaii_70_2019_6B.1A.5a.1
A_Tunisia_2034_2020
A_Sergipe_13444_2020
A_Cote_DIvoire_1448_2021
A_Guangdong-Maonan_SWL1536_2019_6B.1A.5a.1
A_Minas_Gerais_12704_2020
A_California_134_2017
A_Macao_604290_2018
A_Brunei_46_2019
A_District_of_Columbia_T0219_2017
A_Belgium_G0157_2018
A_Pakistan_2303_2018
A_Maryland_42_2019_6B.1A.5b
A_Jiangsu-Tinghu_SWL11150_2018
A_shenzhen_2019-14164_2018
A_Paris_1447_2017_6B.1A
A_South_Africa_R06617_2017
A_Mozambique_0218_2018
A_Rio_Grande_Do_Sul_17_2017
A_Michigan_45_2015_6B.1
A_South_Africa_3626_2013_6B
A_Brazil_600118_2016
A_Shanghai-Xuhui_SWL1809_2019
A_California_99_2016
A_Iowa_53_2015_6B.2
A_Vietnam_37613_2016
A_Nepal_527_2016
A_England_235_2016
A_California_08_2016
A_Georgia_26_2016
A_Belize_1097_2016
A_Colombia_9890_2016
A_Iiv-Orenburg_120-L_2016
A_Newcastle_16_2016
A_Mali_089_2016
A_Elephant_seal_California_1_2010
A_elephant_seal_California_2_2010
A_California_07_2009
0.003

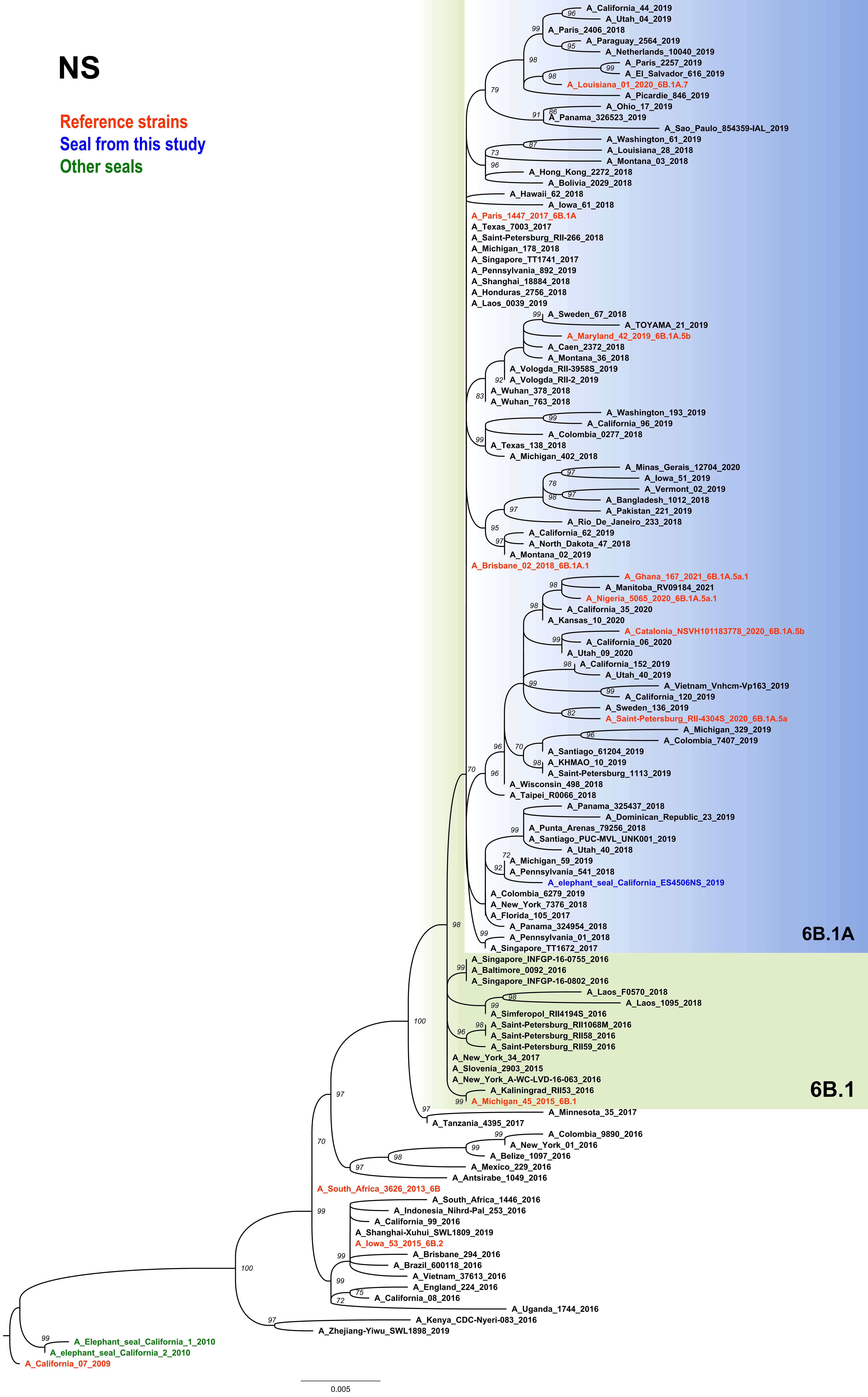
NS
Reference strains
Seal from this study
Other seals
A_California_44_2019
A_Utah_04_2019
A_Paris_2406_2018
A_Paraguay_2564_2019
A_Netherlands_10040_2019
A_Paris_2257_2019
A_El_Salvador_616_2019
A_Louisiana_01_2020_6B.1A.7
A_Picardie_846_2019
A_Ohio_17_2019
A_Panama_326523_2019
A_Sao_Paulo_854359-IAL_2019
A_Washington_61_2019
A_Louisiana_28_2018
A_Montana_03_2018
A_Hong_Kong_2272_2018
A_Bolivia_2029_2018
A_Hawaii_62_2018
A_Iowa_61_2018
A_Paris_1447_2017_6B.1A
A_Texas_7003_2017
A_Saint-Petersburg_RII-266_2018
A_Michigan_178_2018
A_Singapore_TT1741_2017
A_Pennsylvania_892_2019
A_Shanghai_18884_2018
A_Honduras_2756_2018
A_Laos_0039_2019
A_Sweden_67_2018
A_TOYAMA_21_2019
A_Maryland_42_2019_6B.1A.5b
A_Caen_2372_2018
A_Montana_36_2018
A_Vologda_RII-3958S_2019
A_Vologda_RII-2_2019
A_Wuhan_378_2018
A_Wuhan_763_2018
A_Washington_193_2019
A_California_96_2019
A_Colombia_0277_2018
A_Texas_138_2018
A_Michigan_402_2018
A_Minas_Gerais_12704_2020
A_Iowa_51_2019
A_Vermont_02_2019
A_Bangladesh_1012_2018
A_Pakistan_221_2019
A_Rio_De_Janeiro_233_2018
A_California_62_2019
A_North_Dakota_47_2018
A_Montana_02_2019
A_Brisbane_02_2018_6B.1A.1
A_Ghana_167_2021_6B.1A.5a.1
A_Manitoba_RV09184_2021
A_Nigeria_5065_2020_6B.1A.5a.1
A_California_35_2020
A_Kansas_10_2020
A_Catalonia_NSVH101183778_2020_6B.1A.5b
A_California_06_2020
A_Utah_09_2020
A_California_152_2019
A_Utah_40_2019
A_Vietnam_Vnhcm-Vp163_2019
A_California_120_2019
A_Sweden_136_2019
A_Saint-Petersburg_RII-4304S_2020_6B.1A.5a
A_Michigan_329_2019
A_Colombia_7407_2019
A_Santiago_61204_2019
A_KHMAO_10_2019
A_Saint-Petersburg_1113_2019
A_Wisconsin_498_2018
A_Taipei_R0066_2018
A_Panama_325437_2018
A_Dominican_Republic_23_2019
A_Punta_Arenas_79256_2018
A_Santiago_PUC-MVL_UNK001_2019
A_Utah_40_2018
A_Michigan_59_2019
A_Pennsylvania_541_2018
A_elephant_seal_California_ES4506NS_2019
A_Colombia_6279_2019
A_New_York_7376_2018
A_Florida_105_2017
A_Panama_324954_2018
A_Pennsylvania_01_2018
A_Singapore_TT1672_2017
6B.1A
A_Singapore_INFGP-16-0755_2016
A_Baltimore_0092_2016
A_Singapore_INFGP-16-0802_2016
A_Laos_F0570_2018
A_Laos_1095_2018
A_Simferopol_RII4194S_2016
A_Saint-Petersburg_RII1068M_2016
A_Saint-Petersburg_RII58_2016
A_Saint-Petersburg_RII59_2016
A_New_York_34_2017
A_Slovenia_2903_2015
A_New_York_A-WC-LVD-16-063_2016
A_Kaliningrad_RII53_2016
A_Michigan_45_2015_6B.1
6B.1
A_Minnesota_35_2017
A_Tanzania_4395_2017
A_Colombia_9890_2016
A_New_York_01_2016
A_Belize_1097_2016
A_Mexico_229_2016
A_Antsirabe_1049_2016
A_South_Africa_3626_2013_6B
A_South_Africa_1446_2016
A_Indonesia_Nihrd-Pal_253_2016
A_California_99_2016
A_Shanghai-Xuhui_SWL1809_2019
A_Iowa_53_2015_6B.2
A_Brisbane_294_2016
A_Brazil_600118_2016
A_Vietnam_37613_2016
A_England_224_2016
A_California_08_2016
A_Uganda_1744_2016
A_Kenya_CDC-Nyeri-083_2016
A_Zhejiang-Yiwu_SWL1898_2019
A_Elephant_seal_California_1_2010
A_elephant_seal_California_2_2010
A_California_07_2009
0.005
