# Supplementary material for: Human influenza A virus H1N1 in marine mammals in California, 2019
Source: PLoS One. 2023 Mar 30;18(3):e0283049. doi: 10.1371/journal.pone.0283049 (PMC10062622; doi:10.1371/journal.pone.0283049)

Supplementary Figure S9. Time-scale Bayesian MCC tree of the H1 gene of 165 A(H1N1)pdm09 isolates sampled globally between 2016–2021. Bayesian posterior probability values > 70% are indicated.

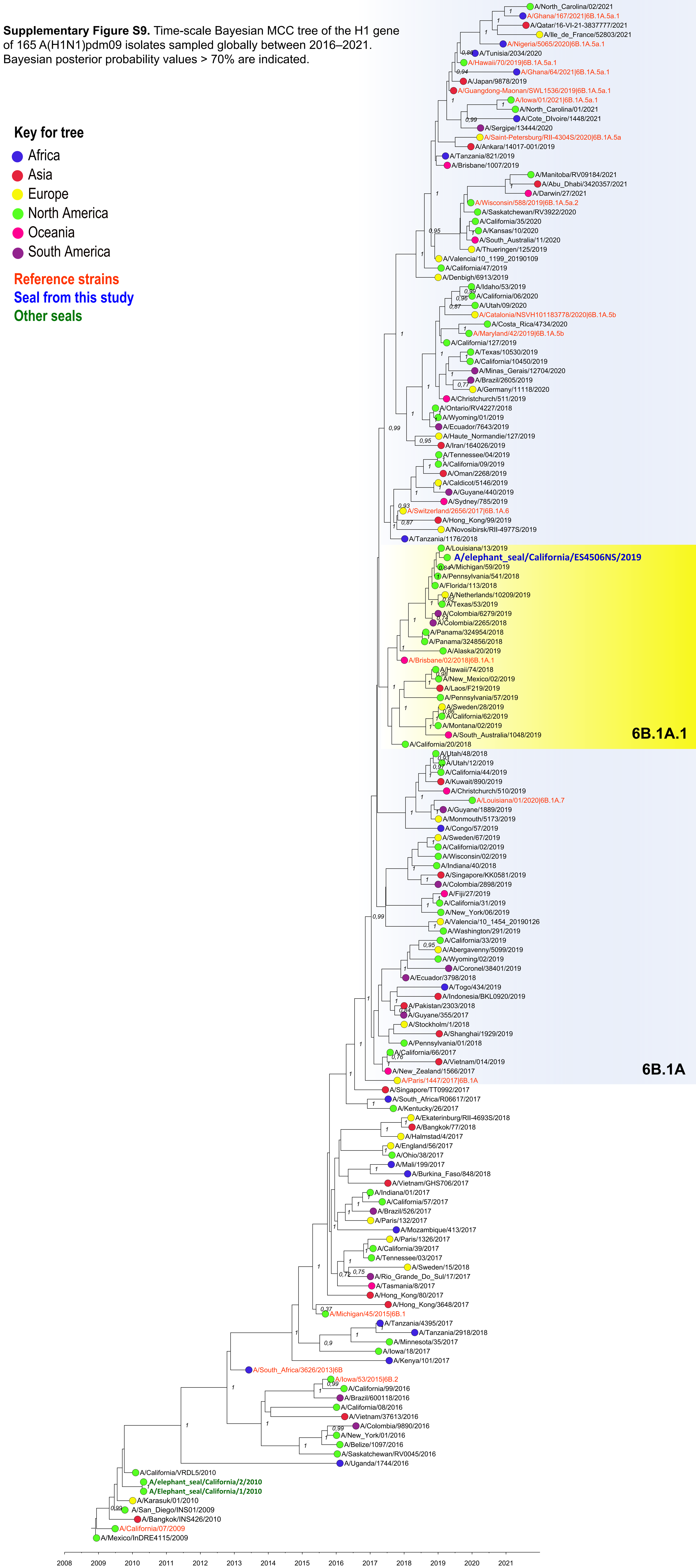

Supplement: S2 File — Bayesian posterior probability values > 70% are indicated. (PDF) [file pone.0283049.s003.pdf]
